# Antioxidant Properties of Amazonian Fruits: A Mini Review of In Vivo and In Vitro Studies

**DOI:** 10.1155/2019/8204129

**Published:** 2019-02-17

**Authors:** Raúl Avila-Sosa, Andrés Felipe Montero-Rodríguez, Patricia Aguilar-Alonso, Obdulia Vera-López, Martin Lazcano-Hernández, Julio César Morales-Medina, Addí Rhode Navarro-Cruz

**Affiliations:** ^1^Departamento de Bioquímica-Alimentos, Facultad de Ciencias Químicas, Benemérita Universidad Autónoma de Puebla, Puebla, Mexico; ^2^Research Center for Animal Reproduction, CINVESTAV-UAT, Tlaxcala, Mexico

## Abstract

Brazil, Colombia, Ecuador, Peru, Bolivia, Venezuela, Suriname, Guyana, and French Guiana share an area of 7,295,710 km^2^ of the Amazon region. It is estimated that the Amazonian forest offers the greatest flora and fauna biodiversity on the planet and on its surface could cohabit 50% of the total existing living species; according to some botanists, it would contain about 16-20% of the species that exist today. This region has native fruit trees in which functional properties are reported as antioxidant and antiproliferative characteristics. Amazon plants offer a great therapeutic potential attributed to the content of bioactive phytochemicals. The aim of this mini review is to examine the state of the art of the main bioactive components of the most studied Amazonian plants. Among the main functional compounds reported were phenolic compounds, unsaturated fatty acids, carotenoids, phytosterols, and tocopherols, with flavonoids and carotenoids being the groups of greatest interest. The main beneficial effect reported has been the antioxidant effect, evaluated in most of the fruits investigated; other reported functional properties were antimicrobial, antimutagenic, antigenotoxic, analgesic, immunomodulatory, anticancer, bronchodilator, antiproliferative, and anti-inflammatory, including hypercholesterolemic effects, leishmanicidal activity, induction of apoptosis, protective action against diabetes, gastroprotective activity, and antidepressant effects.

## 1. Introduction

In the vicinity of the Amazon River, a large number of plants grow. Many of them are slightly known by a large part of the population living in this region. Most of these plant fruits not only are edible but are potentially functional with a variety of beneficial compounds to health. Basically, the problem with native Amazonian fruits is summarized in poor processing technologies and ignorance of their functional compounds and that outside the region, we have very little knowledge of it. The aim of this mini review is to examine the state of the art of the main bioactive components of the most studied Amazonian plants.

## 2. Methodology

Studies with original data related to the presence of functional activity compounds in Amazonian plants (published between 1999 and 2018) were identified by searching electronic databases and reviewing citations. Among the databases were Elsevier, ScIELO, Dialnet, and Redalyc, including publications in English, Spanish, and Portuguese. Eligible studies for this review included randomized controlled trials in humans, experimental animals, or cell cultures, with prospective, parallel, or crossed designs, with full text, and whose results showed a protective effect against oxidative stress and/or favorable effects on some pathological conditions. There was no restriction on the type of publication or sample size. Documents whose main information was related to technological processing or could not verify the functional effects of Amazonian plants were excluded.  Table 1 shows the main compounds and the biological activities reported for the plants reviewed.

## 3. Monographs

### 3.1. *Eugenia stipitata* (McVaugh, 1956)

**Figure 1:**
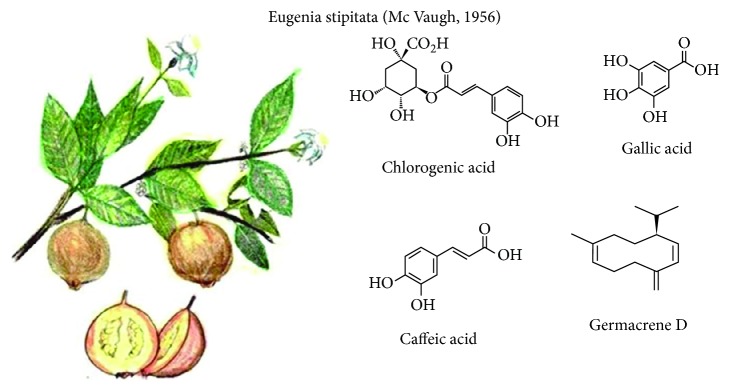
*Eugenia stipitata* McVaugh and its main compounds with functional activity.

Also known as quince, Amazonic guava, arazá, or ara*ς*a in Brazil, it is a climacteric fruit of the Myrtaceae family from the Ecuadorian Amazon region. It grows in deep, fertile, and well-drained soils. It is harvested from 38 days of the transition from flower to fruit with a frequency of three crops per year. It has an oval shape (Figure 1), with a longitudinal diameter and transverse diameter of 5-10 cm and 7-8 cm, respectively, with a yellow pulp and skin and an average weight of 150 g (per fresh fruit). Its epicarp is thin, with fine pubescence and light-green color that turns yellowish or orange at maturity [1, 2].

It has a moisture content of 82-83% and an acidic taste (pH~2.5). It is a delicate and easily decomposable fruit; the postharvest shelf life is shortened as a result of anthracnose and other decay problems [3]. Protein content and minerals are 11.9 ± 0.5 and 4 ± 0.1, respectively (dry base); soluble sugar content represents around 50% of the dry weight being its main constituent fructose. Total dietary fiber content is high compared to other tropical fruits, reaching about 35% (d/w) [2]. It is rich in volatile terpenes, fiber, and mainly vitamin C. Some studies have shown antioxidant activity and a high phenolic content that differ between different arazá genotypes. Arazá fruit revealed a total phenolic quantity of 184.05 ± 8.25 mg of gallic acid/100 g of extract with antioxidant activity. No cytostatic effects have been demonstrated; however, antimutagenic and antigenotoxic activities were observed at doses of 300 mg of extract/kg of body weight; so, *E. stipitata* could contribute to antimutagenic and antigenotoxic activities [4, 5].

A greater antioxidant activity is showed in the green state. As maturity degree advances, especially in the epicarp, chlorogenic, gallic, and caffeic acids are the major phenols responsible for antioxidant activity [6, 7]. Among the identified carotenoids, lutein and esters with palmitic and myristic acids were identified: lutein dipalmitate, lutein palmitate-myristate, *β*-cryptoxanthin palmitate, and zeaxanthin palmitate [6]. Essential oils present in tree leaves showed a complex pattern of monoterpenes and sesquiterpenes (69.5%), of which approximately 52% of them being oxygenated. One of these molecules, Germacrene D, could be responsible for the cytotoxic activity on the HCT116 human colon carcinoma cell line [8, 9], as well as its antimicrobial capacity [10].

### 3.2. *Euterpe oleracea* (Mart, 1824)

**Figure 2:**
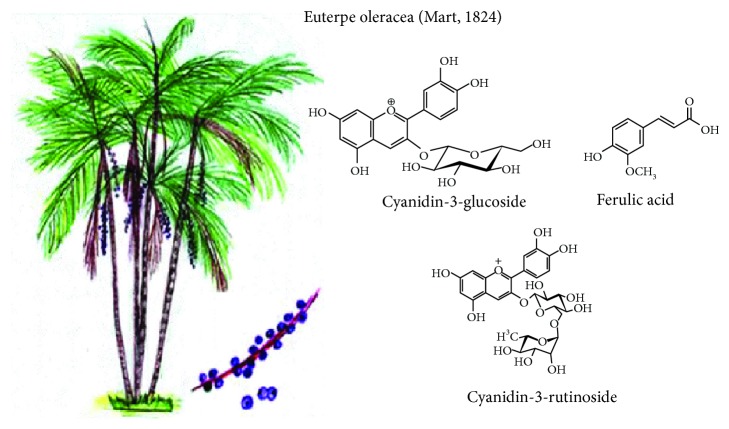
*Euterpe oleracea* Mart and its main compounds with functional activity.

It is a widespread palm tree, with an incidence and economic importance in the Amazonian delta flood plains, known by the names of palm of asaí, azaí, huasaí, palma murrapo, naidí, or generally acai. The fruit is produced in clusters from a third-year growth. Each fruit (Figure 2) is a sessile stone fruit with a woody endocarp, round shape, 1-2 cm diameter, and mass that varies from 0.8 to 2.3 g. Its fruits are constituted by a slightly hard seed, surrounded by a greyish and oily pulp, covered by a dark-purple epidermis [11, 12]. Fruits and roots are traditionally being used against diarrhea, jaundice, skin complications (acne, eczema), and parasitic infections (helminths) and as a remedy against influenza, fever, and pain [13].

The polyphenolic profile and antioxidant activity of Colombian acai are different from the one carried out with several Brazilian acai studies. Colombian acai has higher proportions of delphinidin, cyanidin (cyanidin-3-glucoside), and ferulic acid with high antioxidant activity [14, 15]. Proanthocyanidins were detected from acai seed aqueous extract, as well as their bioactivity (antioxidant and cytotoxic activities) depending strongly on their phenolic profile. However, other nonphenolic compounds may be involved in their antioxidant activity [16]. Moreover, in healthy women, it has been observed that the consumption of acai pulp improves the concentration of antioxidant cellular enzymes and serum biomarkers increasing catalase activity, total antioxidant capacity, and the reduction of reactive oxygen species and carbonyl protein concentration [17].

Among other studies, acai showed antiparasitic activity against *L. infantum* and *L. amazonensis* without cytotoxic effects to the host cell [18], reduction of early carcinogenesis in the colon of mice, mitigation of DNA damage induced by azoxymethane [19], antitumorigenic potential in the MCF-7 cell line [20], reduction in selected markers of metabolic disease risk in overweight adults [21], protection against renal damage in diabetic rats [22], inhibition of urinary bladder carcinogenesis in mice [23], improvement of cardiac dysfunction and exercise intolerance in rats subjected to myocardial infarction [24], and prevention of oxidative damage in the brain of rats [25].

### 3.3. *Myrciaria dubia* (HBK) (McVaugh)

**Figure 3:**
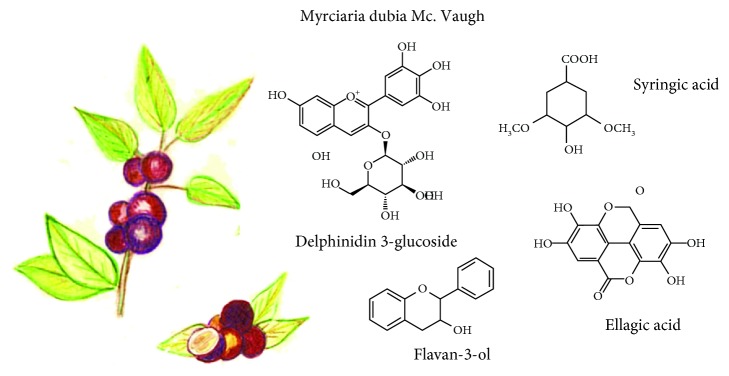
*Myrciaria dubia* McVaugh and its main compounds with functional activity.

Its common names are camu-camu, caçari, arazá de agua, guayabo, guayabito, or guapuro blanco. It grows near the river and lake margins (Figure 3). Its high phenolic and vitamin C concentration contributes to a high antioxidant capacity and the consequent health benefits [26, 27].

Camu-camu is a spherical fruit (with a diameter and length of approximately 1.0-3.2 cm and 1.2-2.5 cm, respectively) [28]. Polyphenolic compounds, antioxidant concentration, and antioxidant capacity depend on their maturity state [28]. Before harvest, carotenoids, flavonoids, and anthocyanins, as well as vitamin C, are in higher concentrations. When the fruit ripens, ascorbic acid concentration decreases, while anthocyanin, flavonol, and flavanol content, as well as the antioxidant capacity, increased [26, 29].

Chemical analysis by HPLC identified the presence of catechin, delphinidin 3-glucoside, cyanidin 3-glucoside, ellagic acid, and rutin. Other phenolic compounds were also present such as flavan-3-ol, flavonol, flavanone, and ellagic acid derivatives. Acid hydrolysis of phenolic fraction revealed the presence mainly of gallic and ellagic acids, which suggests that this fruit has important quantities of hydrolyzed tannins (gallotannins and/or ellagitannins) [29].

It has been observed in rats with diabetes type 1 that camu-camu frozen pulp extracts attenuate hyperlipidemia and lipid peroxidation. This could be due to the presence of flavonoids such as quercetin and myricetin that would be contributing to avoid oxidative damage, relieving diabetic complications in this animal model [30]. Camu-camu juice has an antigenotoxic effect in acute, subacute, and chronic treatments in blood cells of mice. This effect is being observed only in ex vivo studies, with more significant results in juice acute administration, without toxic effects or posttreatment death [31]. Moreover, compounds such as ellagitannins, ellagic acid, quercetin glucosides, syringic acid, and myricetin could be the main reason for a protection effect against microvascular complications (associated with diabetes type 2) and against some bacterial infections; *in vitro* evaluation showed antihyperglycemic, antihypertensive, antimicrobial, and cell rejuvenation activities [32]. Camu-camu residues have also demonstrated antioxidant, antimicrobial, and antienzymatic activities [33].

### 3.4. *Solanum sessiliflorum* (Dunal, 1814)

**Figure 4:**
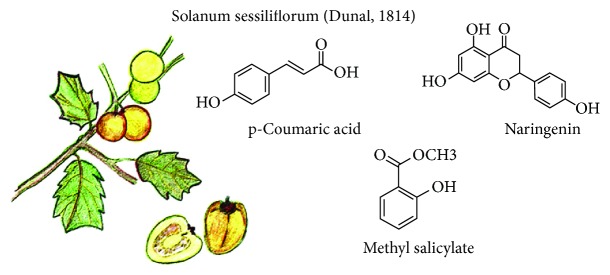
*Solanum sessiliflorum* Dunal and some compounds with functional activity.

Its name is cocona and it is an herbaceous shrub whose fruits vary from almost spherical or ovoid to oval. With a 4 to 12 cm width and a 3 to 6 cm length and a 240 to 250 g weight, it has a color from yellow to reddish (Figure 4). Their hull is soft and is surrounded by a thick, yellow, and watery mesocarp; it has an unusual taste, highly acid. It is consumed in salads and juices [34]. Cocona is slightly known mainly due its small-scale production [35]. However, local population consumes it very frequently as hypocholesterolemic and hypoglycemic remedies and for skin disease treatment [36, 37].

Its components include the presence of p-coumaric acid, p-hydroxydihydrocoumaric acid, naringenin, methyl salicylate, long-chain hydrocarbons, fatty acids, and their methyl and ethyl esters. Some of these compounds accumulate only in fruit epicarp. Chromatographic profile comparison between volatile compounds and different morphotypes (oval, small round, and large round) showed chemical differences; the oval morphotype exhibits greater chemical complexity in terms of volatile and nonvolatile metabolites. Furthermore, cytotoxic, genotoxic, and antigenotoxic potential was evaluated *in vivo*, observing a noncytotoxic effect on bone marrow cells and a nongenotoxic effect on Wistar rats. Cocona antioxidant capacity may contribute to the antigenotoxic effects [38, 39]. Finally, cocona flour administration showed a reduction of total cholesterol concentration, low-density lipoprotein (LDL-c), and liver cholesterol and increasing cholesterol and high-density lipoprotein (HDL-c) fecal excretion in hypercholesterolemic rats [40].

### 3.5. *Theobroma grandiflorum* (Willd. ex Spreng.) (K. Schum, 1886)

**Figure 5:**
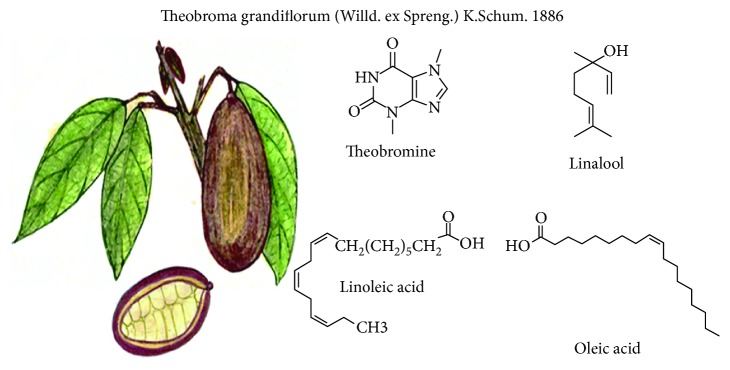
*Theobroma grandiflorum* and its main compounds with functional activity.

It is a tree that reaches 15-20 m high; it belongs to the *Sterculiaceae* family [41]. Its fruits have different shapes (oblong, round oval), weighing between 200 g and 4000 g (Figure 5) [42]. It is known as copoazú and belongs to the Theobroma genus, like cocoa, and is considered as one of the most popular fruits in the Amazonian market [43].

From copoazú almonds, it is obtained as a cocoa-like liquor, with improved characteristics on unsaturated fatty acid percentages and a smooth and pleasant flavor. It has active antioxidant substances, low percentage of theobromine, and high content of linoleic and oleic unsaturated fatty acids. It is considered a suitable product for cosmetic, chocolate, beverage, liquor, and food industries [44]. Its main components in pulp were detected such as volatile compounds: 24 esters, 13 terpenes, 8 alcohols, 4 carbonyls, 4 acids, 2 lactones and phenol, ethyl butanoate, ethyl hexanoate, and linalool [41].

Polyphenols derived from copoazú were studied evaluating the distribution and metabolism in the gastrointestinal tract of mice and the microbial metabolic conversion of a unique combination of flavonoids (flavan-3-ols, procyanidins, and flavones). These compounds are accumulated mainly in the stomach and small intestine where they could exert local effects. Procyanidin microbial metabolism was different from cocoa that contains procyanidin too [43]. Further, copoazú and cocoa liquors were chronically provided to diabetic rats with streptokin. Copoazú liquor improves their lipid profile and antioxidant status, which could suggest a superior effect of the cocoa liquor [45].

### 3.6. *Mauritia flexuosa* L.f. (1782)

**Figure 6:**
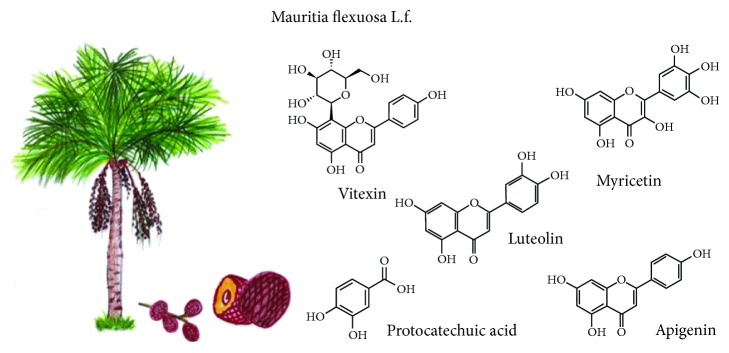
*Mauritia flexuosa* L.f. and some compounds with functional activity.

It is commonly known as canangucha, buriti, or moriche palm. It is considered the most abundant native palm that grows naturally in the Brazilian Amazon biome. Its fruit is highly nutritious with a yellow-orange pulp (Figure 6) and bittersweet taste. Its endocarp is surrounded by a spongy material made of starch and oil, with a hard skin, and contains a small reddish-brown scale-like fruit [46]. It is possible to extract oil from its pulp, whose main components are palmitic (18.7%), stearic (1.5%), oleic (76.7%), linoleic (1.5%), linolenic (0.7%), and arachidic acid (0.5%) [47].

The moriche plant has phenolic compounds mainly flavonoids and glycosylated anthocyanins like the following: catechin, caffeic acid, chlorogenic acid, quercetin, naringenin, myricetin, vitexin, scoparin, rutin, cyanidin-3-rutinoside, cyanidin-3-glucoside, epicatechin, and kaempferol [48]. On the other hand, the fruits show a reasonable amount of phenolic compounds, carotenoids (with predominance of *β*-carotenes), and antioxidant activity, which confirms the functional potential of moriche [49]. Fruit pulp extracts showed six phenolic acids: p-coumaric, ferulic, caffeic, protocatechuic, chlorogenic, and quinic. Quinic acid is much more abundant than other phenolic acids in pulp; extracts also show seven kinds of flavonoids such as catechin, epicatechin, apigenin, luteolin, myricetin, kaempferol, and quercetin [50]. In leaves, tricine-7-O-rutinoside, apigenin-6-C-arabinoside, 8-C-glucoside (isoschaftoside), kaempferol-3-O-rutinoside (nicotiflorine), quercetin-3-O-rutinoside (rutin), luteolin-8-C-glucoside (orientin), and luteolin-6-C-glucoside (isoorientin) were identified [51]. Leaf extract revealed its great ability to inhibit food pathogens, like *Pseudomonas aeruginosa*, and a moderate antimicrobial activity when applied in fruits [48].

### 3.7. *Plukenetia volubilis* L.

**Figure 7:**
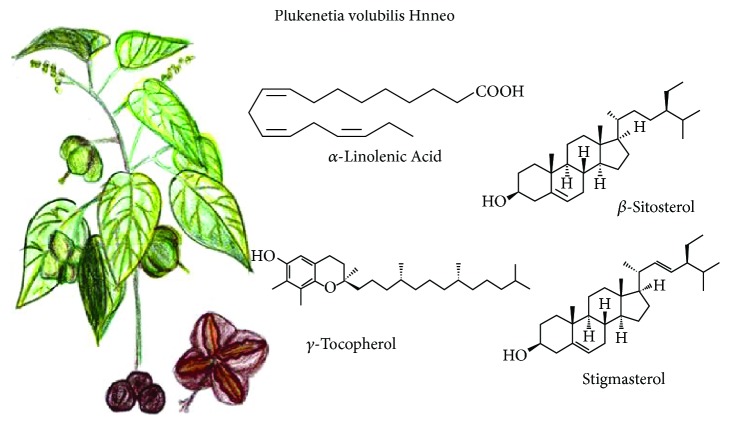
*Plukenetia volubilis* L. and its main compounds with functional activity.

It is a domesticated grapevine known also as sacha inchi, sacha yuchi, sacha yuchiqui, mountain peanuts, wild peanuts, or inca peanuts among others. It grows in warm climates, at high altitude in the Andean rainforest to the Peruvian Amazon lowlands (Figure 7). Due to its oil content, it is used as food supplement, in skin care, and for wound treatment, insect bites, and skin infections [52].

The most studied and interesting fraction of this fruit is its oil. Fruit seeds are a suitable oil source (35-60%) rich in omega 3 and 6, whose composition varies according to seed varieties. Found were significant contents of *α*-linolenic acid and a low linolenic acid/linoleic acid ratio, as well as considerable amounts of tocopherols (*γ*- and *δ*-tocopherols), phytosterols (*β*-sitosterol and stigmasterol), and phenolic compounds like ferulic acid. However, no correlations have been found between hydrophilic and lipophilic bioactive compounds and antioxidant capacity. It suggests a complex interaction of different antioxidant compounds with different action modes. Although there are few studies on the sacha inchi oil effects on health, there are evidences that it could act by improving the lipid profile [52]. Regarding its use for skin care, sacha inchi oil was very active as a nonstick (preventive) in keratinocytes and in the detachment of *Staphylococcus aureus* on the adherence to *in vitro* human skin explants [53].

### 3.8. *Bactris gasipaes* H. B. Kunth

**Figure 8:**
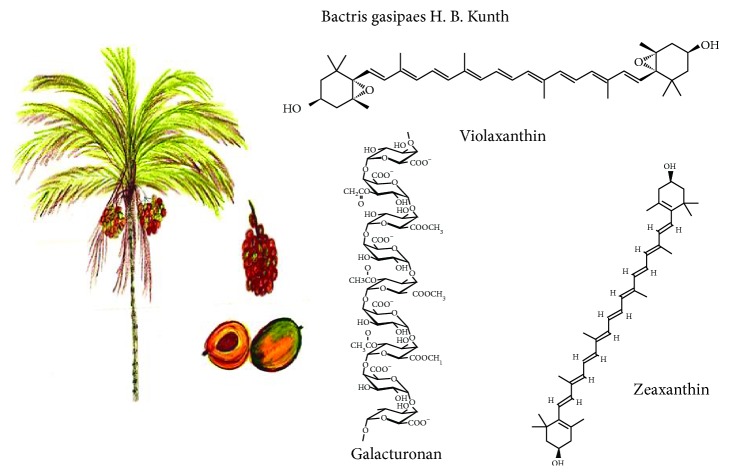
*Bactris gasipaes* H. B. Kunth and its main functional compounds.

It is an Amazonian palm grown mainly for fruit production (Figure 8), known as chontaduro, pejiballe, acana, or pupunha [54]. The chontaduro fruit has considerable concentration of proteins and oil [55], with an important content of linoleic and linolenic polyunsaturated fatty acids [56], as well as *β*-carotenes [57].

Chontaduro flour residues contain different types of carotenoids: violaxanthin, lutein, zeaxanthin, 15-cis *β*-carotene, 13-cis *β*-carotene, all-trans *β*-carotene, 9-cis *β*-carotene, and *α*-carotene, as the main carotenoid pigment. Retinol equivalent values found for chontaduro cooked fruit (traditional consumption form) and flour are higher than those reported for popular products such as tomato and papaya [58, 59]. Chontaduro flour carbohydrates are predominantly composed of insoluble fiber, highly esterified homogalacturonan (70% of esterification). It contains linear methyl and minor portions of xylogalacturonan and rhamnogalacturonan that may promote health benefits. Although not very well documented in the literature, probably refer to their antioxidant capacity and their nutritional value since their protein contains eight essential amino acids [60].

### 3.9. *Paullinia cupana* Kunth (1823)

**Figure 9:**
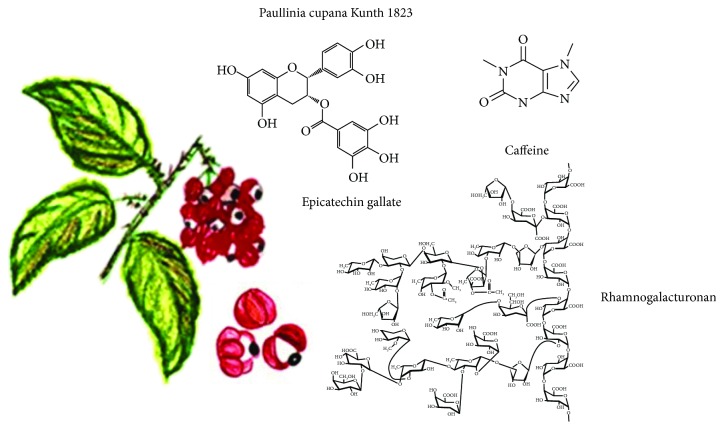
*Paullinia cupana* Kunth, 1823, and some functional compounds.

This climbing shrub, better known as guarana, is rich in vitamins and stimulants such as caffeine; so, it is used mainly for consumption as beverage (Figure 9). It is produced mainly in the Brazilian states of Amazonas and Bahia, and approximately 70% of its production is used in soft and energy drink industries [61]. Its seeds are used to produce guarana powder, which is consumed mainly due to its stimulating activity [62]. The main reason so far to study guarana is its caffeine content, and this probably will continue due to the high demand of this alkaloid in the pharmaceutical and cosmetic industries. Semipurified guarana extract shows antidepressant and panicolytic effects [63]. Guarana seed extracts present antimicrobial activity against *Escherichia coli*, *Pseudomonas fluorescens*, *Bacillus cereus*, and spoilage fungi such as *Aspergillus niger*, *Trichoderma viride*, and *Penicillium cyclopium* [64].

All guarana seed extracts have antioxidant activity with high amounts of total phenolic compounds like catechins, such as epicatechin, catechin, and epicatechin gallate. Due to their high antioxidant, antibacterial, and antifungal activities, guarana extracts have a promising potential as natural antioxidants in food, cosmetic, and pharmaceutical industries [64].

The presence of dietary fiber, including pectic and hemicellulose polysaccharides has been reported, and a homogalacturonan with rhamnogalacturonan and xylans has also been isolated and characterized. Pectic polysaccharides and methanolic extract exhibited antioxidant activity, and part of the possible antioxidant effects of guarana could be attributed to their pectic component [62].

## 4. Antioxidant Capacity of Native Amazonian Fruits

In summary, most of the compounds with functional activity correspond to compounds with antioxidant activity; Table 2 shows the different methods used in the references examined in the present mini review. However, the comparisons are extremely complicated so it would be more appropriate to review clinical studies performed on animals, but unfortunately, to date, there are very few of them. The antioxidant capacity methods were DPPH, FRAP, TEAC, ABTS, and ORAC. Comparison of antioxidant capacity between fruits should be made only when the conditions (method, solvent, sampling, expression of results, etc.) analyzed are the same; therefore, results are not comparable with a great disadvantage that presents to compare the antioxidant capacities of various fruits.

## 5. Conclusion

According to numerous authors, many Amazonian fruits are an adequate source of multiple compounds with potential health benefits, mainly antioxidant effects, which has also been proven through numerous studies such as those detailed in this mini review. However, among its differences in composition, quality, and insufficient in vivo tests, scientific evidence offers challenges and great opportunities in different areas of research (toxicology, food safety, food technology, and processing). Therefore, new trends in functional foods should be conducted considering the enormous potential of these Amazonian fruits in human health.

## Figures and Tables

**Table 1:** Biological activity and main responsible compounds of some Amazonian plants.

Botanical name	Functional compounds	Functional properties	Reference
*Eugenia stipitata*	Phenolic compounds (chlorogenic, gallic, and caffeic acids), carotenoids (xanthophylls and carotenes)	Antioxidant, antimutagenic, and antigenotoxic	[5, 7, 15]
*Euterpe oleracea*	Phenolic compounds (flavonoids) and carotenoids	Antioxidant, leishmanicide, antimicrobial, immunomodulatory, and antigenotoxic	[14, 16, 18, 19],
*Myrciaria dubia*	Phenolic compounds (flavonoids), carotenoids, and vitamin C	Antioxidant, antimicrobial, and antigenotoxic	[26–29, 31, 32]
*Solanum sessiliflorum*	Ascorbic acid, p-coumaric acid, p-hydroxy dihydro coumaric acid, naringenin, methyl salicylate, long chain hydrocarbons, fatty acids, and their methyl and ethyl esters	Antioxidant, hypocholesterolemic, and antigenotoxic	[39, 40]
*Theobroma grandiflorum*	Theobromine, volatile compounds (aldehydes, ketones and alcohols, ethyl butanoate, ethyl hexanoate, and linalool), unsaturated fatty acids, and flavonoids	Antioxidant, probiotic, and reduction of hypertriglyceridemia	[42, 43, 45, 47]
*Mauritia flexuosa*	Phenolic compounds (phenolic acids and flavonoids) and carotenoids	Antioxidant and antimicrobial	[46, 48, 49]
*Plukenetia volubilis*	Polyunsaturated fatty acids, tocopherols, phytosterols, and phenolic compounds	Antioxidant	[52, 53]
*Bactris gasipaes*	Unsaturated fatty acids (oleic, linoleic, and linolenic), carotenoids (*β*-carotene, lutein, zeaxanthin, *β*-cryptoxanthin, and *α*-carotene), and dietary fiber	Antioxidant, precursor of vitamin A	[54–60]
*Paullinia cupana*	Phenolic compounds: catechin, epicatechin, and proanthocyanidins; also dietary fiber, theobromine, theophylline, and caffeine	Antioxidant, stimulant, antimicrobial, antihyperglycemic, and cytoprotective effect	[62–64]

**Table 2:** Antioxidant capacity of native Amazonian fruits.

Name	DPPH	ORAC	ABTS	FRAP	Reference
*Eugenia stipitata*	IC 500.69 ± 0.23 *μ*g/mL	371.98 ± 11.50 *μ*mol TE/100 g DW	N/R	N/R	[5]
*Euterpe oleracea*	21,049 ± 3,071.0 *μ*mol TE/100 g DW; 12,420 *μ*mol TE ± 465/100 g DW; 609.1 *μ*mol TE/g DW IC50 : 8.8 ± 0.27 *μ*g/mL	101,336.1 *μ*mol TE/100 g DW; 686.0 *μ*mol TE/100 g DW	24.7 ± 10.6 *μ*mol TE/100 g DW; 40,330 ± 19,656 *μ*mol TE/100 g DW	3,834 ± 56 mg ascorbic acid/100 g DW	[6, 14, 16]
*Myrciaria dubia*	185 ± 11 (*μ*mol TE/g FW); 1,679 ± 75 (*μ*mol TE/g DW); 2,138.7 *μ*mol TE/g DW IC 50 1,116.87 ± 0.064 *μ*g/mL	1,002 ± 27 (*μ*mol TE/g DW; 3,060.8 *μ*mol TE/g DW	N/R	N/R	[26, 30, 31]
*Solanum sessiliflorum*	N/R	N/R	N/R	N/R	
*Mauritia flexuosa*	IC 50 19.58 ± 0.064 mg/mL	N/R	33.02 *μ*mol TE/g FW	280.80 ± 37.99 *μ*mol FeSO 4·7H_2_O equiv/100 g	[48, 49]
*Theobroma grandiflorum*	1,913 ± 228 *μ*mol TE/100 g DW	13,628 ± 184 *μ*mol TE/100 g DW	N/R	N/R	[51]
*Plukenetia volubilis*	N/R	6.5 – 9.8 *μ*mol TE/g	N/R	N/R	[29]
*Bactris gasipaes*	N/R	N/R	N/R	N/R	
*Paullinia cupana*	IC50 = 8.5 *μ*g/mL (approximate value taken from the graph)	N/R	N/R	N/R	[63]

TE: Trolox equivalent, VCE: vitamin C equivalent, DW: dry weight; FW: fresh weight; NR: not reported
